# Demonstrator training needs to be active and focused on personalized student learning in bioscience teaching laboratories

**DOI:** 10.1002/2211-5463.13299

**Published:** 2021-09-27

**Authors:** Raheela Awais, Elliott Stollar

**Affiliations:** ^1^ School of Life sciences University of Liverpool UK

**Keywords:** Demonstrators, expectations, first‐ and second‐year undergraduates, laboratory, training, student satisfaction

## Abstract

Demonstrators spend significant time with students on a weekly basis in instructional laboratories and are well poised to offer students meaningful learning. Most often, effective demonstrator training is neglected due to time and resource restraints and it is clear more attention is needed. We hypothesized that students’ learning experience in laboratories would improve if demonstrators were well trained particularly across three overlapping learning domains: subject‐specific knowledge (cognitive and psychomotor), problem solving (cognitive) and group management including personalized student learning strategies (affective). We assessed both students and demonstrators on the impact of this extensive demonstrator training in 1st‐ and 2nd‐year bioscience practical courses over two years. The results show that all students rated the demonstrators’ performance higher after the extensive training. Students from both years valued the provision of problem‐solving skills; however, 1st‐year students placed greater value on the demonstrator’s ability to address student inclusivity, whereas 2nd‐year students preferred the provision of strong subject knowledge. Interestingly, demonstrators’ own perception of their teaching ability was different from student feedback on their performance, which may be due to lack of reflective practice. We propose a multimodal training framework that includes inclusivity/approachability and reflection as an integral part of training. This study further suggests that demonstrator training needs to be tailored to the changing needs of students as they progress through the different levels of their degree. Our proposed framework is particularly relevant to the current pandemic which has affected young people’s mental health, confidence and openness to new experiences.

## Graphical abstract

We ask, if we focus equally on active training of demonstrators with respect to the affective, cognitive and psychomotor learning domains, how will students and demonstrators respond? We found that this training approach was extremely effective and helped us develop an active demonstrator training framework that underscores the importance of personalized student learning and continued demonstrator self‐reflection.

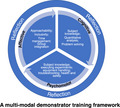

**Abbreviations**
COVID‐19: Coronavirus Disease 2019
GTA: Graduate Teaching Assistants G
SET: Student Evaluation of Teaching
SEEQ: Student Evaluation of Educational Quality

Teaching laboratories for practical‐based courses are a unique place for students to actively apply the concepts and methods covered in theory courses and are therefore essential learning outcomes in Bioscience at the University of Liverpool [1], the UK [2], the United States and beyond [3]. These experiences help science students master team‐based research, reach the highest degrees of self‐reflection and confidence and prepare students for the diverse workforce. Well‐designed laboratory activities allow students to engage with three principal overlapping learning domains based on Bloom’s taxonomy [4, 5]. The cognitive domain for learning reflects students’ knowledge and thinking skills, and the psychomotor domain focuses on students’ sensory awareness to perform manual tasks that require the manipulation of objects or apparatus. The affective domain, on the other hand, reflects students’ ability to change attitudes, beliefs, emotions and feelings [4, 5, 6]. However, engaging in these learning domains requires specialized teaching skills which makes the laboratory a complex teaching and learning environment [3]. For a given laboratory, postgraduate research students often referred as laboratory demonstrators or graduate teaching assistants (GTA), are recruited who fulfil teaching roles in the undergraduate laboratory and facilitate students’ learning in small groups[7, 8]. Demonstrators often come with minimal or no prior experience either as a student in the particular teaching laboratory or as an instructor in this dynamic learning environment [9], and furthermore, effective demonstrator training is frequently neglected usually due to budget and time restraints [10, 11, 12]. It is not surprising that students find their teaching inconsistent and struggle to learn from these inexperienced teachers [13, 14]. Managing and improving demonstrator’s teaching effectiveness is closely linked with students’ experience and satisfaction in instructional laboratories [15]. Furthermore, demonstrators can have a profound impact on students’ experience and for first year students, in supporting them settle into university life, which can be a launchpad for meaningful learning, retention and success [16]. In order to equip our demonstrators to cope with the impact of such challenges, the effectiveness of the development opportunities provided to them needs to be reviewed [17].

## First‐year students’ needs in the lab

First‐year students entering into the higher education are considered to be in the critical transition stage [18]. They come from diverse socio‐economical, cultural and academic backgrounds and are in the process of finding their identity, developing self‐awareness and self‐direction [19]. Failure to handle this transitioning phase adequately can result in significant distress and increased attrition rates [20]. Research has shown that first‐year students are more likely to withdraw from the studies than in the subsequent years [21]. Their first contacts at this stage help establish their aspirations and expectations about university and high‐quality academic as well as nonacademic support plays an important role in the success of first year students [21]. However, providing effective support to students requires the input and coordination of associated staff across the university. Demonstrators account for most of the contact hours with undergraduate students in the department and as such are the key stakeholders with the greatest influence on students learning [22, 23]. This influence has a pronounced effect on first‐year students in particular who often expect ‘teaching’ at university to be similar to what they had experienced at high school and expect lecturers to be just as caring, supportive, approachable and enthusiastic [20, 24, 25]. Students are unable to differentiate between an experienced academic and the demonstrators who themselves are students. ‘They do not want to know that their teacher is sessional or permanent. All they want is high‐quality teaching and high‐quality subjects’ [15]. They expect demonstrators to act as a learning facilitator by using various student‐centred pedagogical approaches rather than an information provider [26]. As students progress through their first year they start to become more achievement driven, concentrating more on performance to establish their academic identity on entering their second year [27]. In second‐year laboratories, students may be more forgiving towards demonstrator teaching ability as they are either predisposed to this kind of learning environment or are willing to take more responsibility for their learning.

### What can be learnt from previous studies concerning demonstrators training

Some previous studies on demonstrator training provide a context for our study, which we have grouped in the following five areas.

### Maximizing resources is crucial for effective demonstrator training

It is clear there are challenges to provide effective demonstrator training with the resources available in any given academic department. Demonstrators’ attitude to their work has been shown to be negatively affected by the amount of time they are actually paid for and their general working conditions [17]. Demonstrators teaching an introductory biology laboratories have also reported struggling with the right balance between demonstrating and their research commitments [28]. It is clear academic departments must carefully consider ways to maximize resources for demonstrators if their teaching is to be effective, enjoyable and equitable [17].

### The focus of demonstrators’ training has been passive and mainly on cognitive and psychomotor skills

Several studies have provided rich insight into best practices for developing demonstrators’ competencies. Many training approaches were initially stand‐alone, covering general pedagogy approaches which involved introducing various learning theories (guided learning, Bloom’s taxonomy, rote, meaningful learning) and general instructional methods (ways to effectively communicate with students and explain concepts clearly) as well as teaching evaluation and feedback, etc. [29, 30, 31, 32, 33, 34]. Later, studies emphasized that the training should be a continuous process and should incorporate combination of generic teaching skills as well as laboratory‐specific skills [34, 35]. Weekly meetings on laboratory‐specific topics were suggested to cover course content, answers to laboratory questions and problem‐solving exercises and fair grading methods, etc. [17, 34, 35]. However, many of these training approaches were in a seminar style, which tends to promote passive learning from the demonstrators. It is now recognized that since introductory laboratories should teach students scientific enquiry, the traditional ‘transmissionist’ approach must be superseded by the constructivist approach to teach cognitive skills through open‐ended exploration by the learner (inquiry‐based learning) [36, 37]. Similarly, active learning and inquiry‐based training for demonstrators is also deemed important to improve demonstrators’ teaching effectiveness [15, 17, 30, 38]

Experimentation in the laboratory evokes not only Bloom’s taxonomy cognitive learning domain (inquiry‐based learning) but also the overlapping psychomotor and affective domains. However, a survey of studies over the past 4 years shows that the emphasis for training demonstrators is often placed principally on the cognitive and psychomotor domains (Table 1). For example, recent training approaches include content knowledge, facilitation skills and process skills, critical thinking and problem‐solving [9], effective communication, instructional practices (including use of external representations) [28, 39, 40], good preparedness [39], designing and grading assignments, time management [28, 41, 42], peer mentoring and reflection [43, 44]. Some of these studies have considered incorporating some aspects of the affective domain in demonstrators’ training such as good engagement with students, empathetic, understanding the learning environment and group dynamics [28, 39], encouraging participation through ‘wait time’ [45] team working and understanding students’ prior knowledge [9, 40]. However, these approaches are at the group level and except for one study do not consider individual students’ emotions and needs [40] (Table 1). This is a missed opportunity as students’ affective learning experiences have been shown to influence their cognitive and psychomotor learning experiences in first year [8, 46, 47]. Furthermore, positive instructor behaviours have been linked to students’ affective learning experiences, which in turn enhance cognitive learning [48]. Another factor that improves participation, affective and cognitive learning is the rapport developed between students and their instructors. [49]. Therefore, it is important that demonstrators are made aware of the extent and influence of students’ emotions and individual needs in the laboratory and their potential role in promoting positive learning experiences [8]. Flaherty *et al* found that demonstrators were unaware of the students’ needs across the cognitive, psychomotor and emotional domains and identified this as a barrier to developing their teaching effectiveness [8]. It is clear that current studies (Table 1) have not sufficiently explored active learning approaches where demonstrators experience the same challenges as the students (doing the same experiment with role‐playing, etc.) and explored the affective domain at the individual student level.

**Table 1:** Literature survey on demonstrators training from 2017 to 2020. 2021 shifts away from training demonstrators to teaching practicals online.

Selected articles on demonstrators’ training	Training focus/ recommendations	Mapping to Bloom’s Taxonomy	Who was surveyed?	Subject
Graduate teaching assistants’ perceptions of teaching competencies required for work in undergraduate science labs [39]	Effective communication to explain concepts, have good preparedness, have good engagement with students and understand learning environment.	Cognitive, psychomotor, affective at group level	GTAs/faculty/lab‐coordinators	Multidisciplinary Labs
Aligning Perceptions of Laboratory Demonstrators’ Responsibilities to Inform the Design of a Laboratory Teacher Development Program [8]	Evidence‐Align‐Develop approach for training since they find a misalignment of perceived demonstrators’ responsibilities with respect to the 3 Bloom’s learning domains.	Cognitive, psychomotor, affective at group level	GTAs and Students	General chemistry
Strategies for training undergraduate teaching assistants to facilitate large active learning classrooms [9]	POGIL (Process Oriented Guided‐Inquiry Learning) to train GTAs in content knowledge, facilitation and process skills such as teamwork, critical thinking and problem‐solving.	Cognitive, affective at group level	GTAs	Organic chemistry Lecture
TA Marking Parties: Worth the Price of Pizza? [42]	Marking parties to engage GTAs in better marking practices.	Cognitive	GTAs	Computer Science Lecture
A Summer Institute for STEM Graduate Teaching Assistants: Exploring Teaching Perceptions [45]	Train demonstrators in pedagogy before teaching term. Learn ‘wait time’ concept to enhance participation.	Cognitive, affective at group level	GTAs	STEM
Relationship between teaching assistants’ perceptions of student learning challenges and their use of external representations (ER) when teaching acid–base titrations in introductory chemistry laboratory courses [40]	Instructional use of external representations for experimental procedures considering prior knowledge of individual students.	Psychomotor, affective at individual student level	GTAs	Chemistry
Benefits and Challenges of Instructing Introductory Biology Course‐Based Undergraduate Research Experiences (CUREs) as Perceived by Graduate Teaching Assistants [28]	GTAs mentor students to take ownership of their learning. GTAs should focus on both practical teaching methods (e.g. designing effective assessments and grading rubrics) and more effective communication strategies (e.g. how to be empathetic or deal with group dynamic issues).	Cognitive, affective at group level	GTAs	Biology
Utility of a Peer Teaching Mentor to Graduate Teaching Assistants [43]	Graduate peer mentors provide GTAs with additional teaching resources to improve their pedagogy.	Cognitive, affective domain of GTAs	GTAs	Social Sciences
Training Future Faculty in 30 Minutes a Week: A Modular Framework to Provide Just‐in‐time Professional Development to Graduate Teaching Assistants [41]	The training modules include sessions on interacting with students, designing and grading assignments and time management.	Cognitive, affective domain of GTAs	GTAs	Biology

### Evaluation of training is essential for a robust training programme

An increasing number of studies have emphasized that the evaluation of demonstrators’ training should be an integral part of the training strategy. Most of the studies have focussed on surveying the demonstrators on their opinions of the training that took place [8, 14, 26, 28, 34, 39, 40] (also see Table 1). Aleamoni (1999) found that the use of well‐designed and correctly analysed student evaluation of teaching (SET) evaluations can be extremely beneficial for students, staff and institutions [50, 51]. Also, how demonstrators and their training influence SET is equally useful to explore [17] as student satisfaction scores reflect perceived learning by students and therefore makes the data useful to assess the effectiveness of demonstrators teaching and their own learning [51]. Despite its importance, only few research papers have investigated the use of SET to directly test the effectiveness of their demonstrator training methods on students and particularly in‐laboratory settings [8, 15, 16, 38].

### Discipline‐specific training in Biosciences is understudied

There are examples of demonstrators’ training development programmes across different nonbiological disciplines including chemistry, physics, maths, electrical engineering, geosciences, etc. [12, 15, 16, 32, 33, 38, 40, 41]; however, only a limited number of studies are available in the field of biosciences [26, 28, 38] (also see Table 1).

### Demonstrators’ perceptions of their responsibilities

Demonstrators’ understanding and application of pedagogy impacts their development as a teacher [26]. Several studies have explored demonstrators deeply held beliefs about teaching and learning [8, 9, 26, 28, 39, 40, 45] (also see Table 1). There is a problematic misalignment between demonstrators and students in the perceptions of demonstrators’ responsibilities for student learning across cognitive: psychomotor and affective domains [8, 17]. However, studies also suggest that instructors can reconceive their teacher’s identity in a short time when introduced to new learner‐centred pedagogies [26, 52]. It is clear that improving demonstrator training is an ongoing process that must continue to evidence barriers to demonstrator learning, to inform better alignment of role expectations and to develop improved teaching capability that is applicable for different disciplines and student groups [8]. Moreover, the demonstrator beliefs need to be regularly challenged to bring change in their teacher identity [53]. From the start of the training, reconciling their beliefs through reflective practice may be key for affecting change. Reflection with peer support or debriefing after each session by academic can help shift the misaligned perceptions and support demonstrators in their ongoing learning [17, 26].

## The current study investigates active, student‐focussed training across the 3 Bloom’s domains

This study continues the process of evidencing barriers, aligning expectations and developing demonstrators’ teaching capability that promises to provide wider implications to the field. We recognize that for demonstrators to successfully enhance the laboratory learning experience of students across cognitive, psychomotor and affective domains, it is essential that demonstrators’ training has learning objectives guided by these domains. Moreover, when learning tends to be more active and inquiry based in laboratories, the training should also be delivered in an active way. We also recognize that the findings from existing demonstrators’ training programmes cannot be easily extrapolated to the field of bioscience as the students joining this discipline come from a much wider range of academic backgrounds (with or without maths/chemistry/physics). In addition, students joining 1st‐year bioscience have a socioeconomic diversity and therefore demand personalized learning strategies, where the student’s individual needs are carefully considered. This is particularly important as many previous studies on training demonstrators have recommended student‐focussed approaches but have briefly touched on the important topics of student diversity and inclusivity, in many cases due to the limitations of time and resources [28].

To improve two bioscience laboratory courses, we hypothesized that instead of solely passive, basic, content‐based training, demonstrators should be actively trained across three domains for student learning: subject‐specific knowledge (theory and practice for each laboratory), inquiry‐based learning (focused on problem‐solving skills) and group management (facilitating group dynamics and responding to students’ individual needs.). To address this hypothesis, we revamped the demonstrators’ training on first‐ and second‐year practical modules. Both the students and the demonstrators were surveyed to evaluate the changes in the teaching effectiveness of demonstrators pre‐ and post‐training. Open comments from students and demonstrators were used to further explore students’ and demonstrators’ views. We then used this information to make recommendations in the form of a multimodal training framework that will prove useful for other laboratory courses with large sizes and diverse cohorts.

## Research Questions

- If we focus equally on active training of demonstrators with respect to the three Bloom’s learning domains, how will students and demonstrators respond to this in the laboratory? Will this response change from a general year 1 to a more specialized year 2 laboratory course?
- How do demonstrators perceive their teaching abilities before and after receiving this training and how does this correspond to the student perceptions?
- What does our data suggest for best practices in training demonstrators?

## Methods

### Participants

First‐ and second‐year bioscience students enrolled in the School of Life Sciences at the University of Liverpool were recruited in the study. All first‐year students (400‐420 students) take the core experimental skill practical module which runs over two semesters. This module covers introductory practical elements for the range of bioscience programmes in the school (Anatomy, Biochemistry, Zoology, Genetics, etc.). The learning objectives are to level the playing field for basic experimental skills such as using basic scientific equipment, planning and executing experiments, data analysis and evaluation of hypotheses. The module is assessed by a range of online tests, abstract‐writing and in‐laboratory competency tests of practical skills, whereas in second year, only a subset of students (140‐160 students) choose the 6‐week molecular biology practical module and were therefore recruited in this study. This module is more specialized, focusing on key molecular biology topics such as microbiology techniques and cloning. The learning objectives are to prepare students for doing molecular biology in future practicals and their research project, and there is more of an emphasis on problem‐solving and writing as well. The module is assessed by a range of online tests, abstract‐writing and a problem‐solving written tests. For both year 1 and year 2 practicals, although the laboratory manuals do provide traditional step‐by‐step instructions, the emphasis for each week is on inquiry‐based learning. This involves extensive problem‐solving exercises related to their work to explore, actively apply and extend their understanding. Year 2, however, was much more advanced and focused on multiweek investigations. Students from 2018/2019 cohort represent the group who received teaching from the demonstrators with minimal training while 2019/2020 cohort received teaching from demonstrators who were provided extensive training (Table 2).

**Table 2:** Training workshops for demonstrators minimal versus extensive training. The elements of extensive training are mapped across three overlapping Bloom’s learning domains with an equal focus on each domain.

Blooms	Our approach	Minimal training	Extensive training
Cognitive & Psychomotor domain	Subject‐specific knowledge	Attend 1‐h student introductory lecture and have 40‐min introduction to practical from academic and 20‐min practice using equipment (no experiment performed).	Short content introduction from an academic. Actively conduct experiment (execute experiment, equipment usage, technique training, data generation). Standardized notes provided with unified explanations. Interactive discussion on health and safety issues for each week. Discussions of good practice for laboratory record keeping and abstract writing
Cognitive domain	Problem solving	No training provided	In and out of class problem‐based calculations to be completed and interactively discussed before practical. Requires asynchronous training materials to be prepared.
Affective domain	Group management	No training provided	Active role‐playing to encourage student engagement with demonstrator and fellow students (peer‐assisted learning). Go through weekly checklist for all students in their group to help develop strategies for personalized student learning. Help to teach time management via lesson planning by discussing the time each activity should take.

The demonstrators recruited for this study were in second or third year of their PhD programmes in different disciplines across the Faculty of Health and Life Sciences at the University of Liverpool. 30 and 12 demonstrators helped teach the 1^st^‐year core experimental skill practical and the 2nd‐year molecular biology practical module, respectively. Within a given teaching laboratory, there was on average 1 academic staff member present with 4 demonstrators. The demonstrator:student ratio was on average 1 to 14 for both courses. 12 (year 1 practical) and 6 (year 2 practical) demonstrators who delivered teaching for two consecutive years and received minimal as well as extensive training (2018 and 2019) were included to allow comparison between minimal versus extensive training. The number of student respondents was lower in 2018 compared with 2019 as the surveys were distributed online in 2018 while they were distributed in person for 2019 which increased participation significantly.

### Training workshops

Training workshops for demonstrators were provided by academic staff who have had a considerable teaching experience in the instructional laboratories. The training took place weekly, a day before each practical day and was categorized as minimal or extensive. Minimal training consisted of an hour‐long training where demonstrators were given theoretical overview of the practical and demonstration of the equipment which were to be used in practical. In the extensive training, demonstrators were provided with 2‐hour face‐to‐face time that was interactive with additional work to do outside of the laboratory in their own time as shown in Table 2.

For the extensive training, weekly monitoring of demonstrators was undertaken to ensure the training was applied during the practical. Demonstrators had to fill in a checklist that reports on the individual needs for each student their group each week which helped academic staff develop support plans. For example, academic staff were able to allocate ‘floating’ demonstrators to these students to give them 1:1 support and get them back on track. Also, opportunities for changing student groups were explored so better peer‐assisted learning could take place with their new partners. This approach made students feel that a diversity of learning needs was being accommodated and that the practical was inclusive.

### Measures

Students and demonstrators were surveyed at the end of semester 1 using online or printed questionnaires. Teaching quality can be evaluated using the nine identifiable dimensions of Student Evaluation of Educational Quality (SEEQ) [54]. SEEQ was originally used by North American Universities to evaluate undergraduate course instructors and has been slightly modified since [38]. In our training, we did not include assessment and grading so factors 6, 7 and 8 were replaced with valuable feedback, suggested reading and record keeping. Additional factors of confidence and problem‐solving skills were included. Demonstrator’s teaching effectiveness was then evaluated using a) a modified 11‐factor SEEQ questionnaire, which was completed both by students and by demonstrators (Table 3); b) thematic analysis using the open comment section to capture qualitative feedback from students as well as demonstrators. The student responses (positive or negative) were coded to three main themes as shown in Table 4 following the step‐by‐step guide as proposed by Maguire & Brid Delahunt [55].

**Table 3:** Modified version of SEEQ questionnaire. After modification, two versions were created: one was used by students to evaluate demonstrators’ teaching effectiveness and other by demonstrators for self‐evaluation of their teaching ability following minimal or extensive training.

	Student version	Demonstrator version
Facilitate Learning	My demonstrator facilitated my learning and understanding of the subject materials in this course	I facilitated my students’ learning and understanding of the subject materials in this course
Was confident	My demonstrator was well‐prepared and confident in conducting the course	I was well‐prepared and confident in conducting the course
Was enthusiastic	My demonstrator was energized and dynamic in conducting the course	I was energized and dynamic in conducting the course
Clear explanations	My demonstrators’ explanations were clear	My explanations were clear
Taught Problem‐Solving	My demonstrator helped me learn problem‐solving skills	I helped my students learn problem‐solving skills
Encourage participation	Students were invited to express their own ideas and /or question the demonstrator	Students were invited to express their own ideas and /or ask questions
Had individual rapport	My demonstrator had a genuine interest in individual student	I had a genuine interest in individual student
Gave valuable feedback	My demonstrators’ feedback on students’ practical work and data analysis was valuable	My feedback on students’ practical work and data analysis was valuable
Helped record keeping	My demonstrator facilitated good laboratory book writing practice	I facilitated good laboratory book writing practice
Suggested Further Reading	Readings /texts/references suggested by my demonstrator were valuable	Readings /texts/references that I suggested were valuable
Overall satisfaction	Overall, my demonstrator was a good teacher	Overall, I was a good teacher

**Table 4:** The open text responses (positive as well as negative) from 1st‐year (*n* = 70) and 2nd‐year (*n* = 102) students were coded to three main themes‐subject knowledge, problem‐solving and accessibility and inclusivity.

Theme	Representative Open text comments
Subject knowledge	My demonstrator knew the information and explained very well. (Positive)
	He provided useful feedback and allowed to see me room for improvements. (Positive)
	Never recommended any reading material. (Negative)
Problem ‐solving	Knew the maths and explained very well. (Positive)
	My demonstrator struggled with maths and problem solving. (Negative)
	He was not confident about the end of practical questions. (Negative)
Approachability and inclusivity	My demonstrator emailed me prior to lab regarding my support plan. This was really appreciated and put me at ease before arrival. (Positive)
	Brings team together, explains each activity making sure that everyone understands. (Positive)
	He fully understood my personal circumstances and helped me catch after missing first half of the practical, always smiling, and approachable. (Positive)

### Data analysis

The responses from students were collected in the form of online and paper questionnaires, and EvaSys software was used to convert Likert scale responses to the numbers from 1 to 5 with 1 being the most negative response (e.g. strongly disagree) and 5 representing the most positive (e.g. strongly agree). For each question, each Likert value was converted to a percentage, and these were plotted as stacked bar graphs. A Mann–Whitney test was performed to test for significance between the two years. *P* < 0.05 was considered as statistically significant results.

### Ethics

Ethics for this study was granted by the University of Liverpool Research Ethics Committee (approval number 5402). No information on gender, age or demographic factors was requested. Participants were given information sheet, and they signed the consent form prior to their participation. Participants were informed that their participation is voluntary, and their responses would be kept anonymous.

## Results and discussion

### Students’ evaluation of demonstrators’ teaching effectiveness

Students’ evaluation scores are a rich source of data to help improve teaching and learning, and they frequently mirror students’ perceived learning across the cognitive, psychomotor and affective domains [16, 51, 56]. Measuring demonstrators’ teaching quality is equally important because students want a high‐quality, seamless education [15]. Therefore, we focussed on students’ and demonstrators’ evaluation data across 2 years and 2 modules to assess our training approach and address our research questions.

**Fig. 1:**
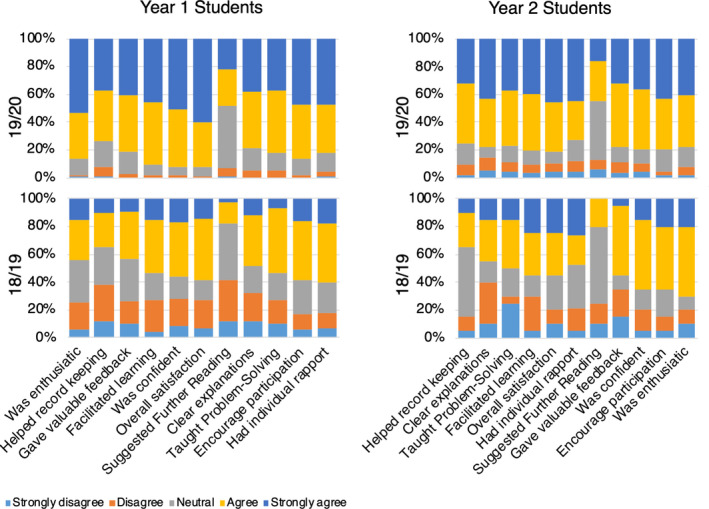
Undergraduate 1st‐ and 2nd‐year students’ evaluation of demonstrators’ teaching effectiveness by 11 factor‐based SEEQ questionnaire following minimal training (201819, *n* = 76) and extensive training (201920, *n* = 283). Vertically paired columns for each factor are significantly different (*P* ≤ 0.001). The % difference between 19/20 and 18/19 for strongly agree and agree categories combined is used to order factors on the graph from highest on the left to lowest difference on the right.

**Fig. 2:**
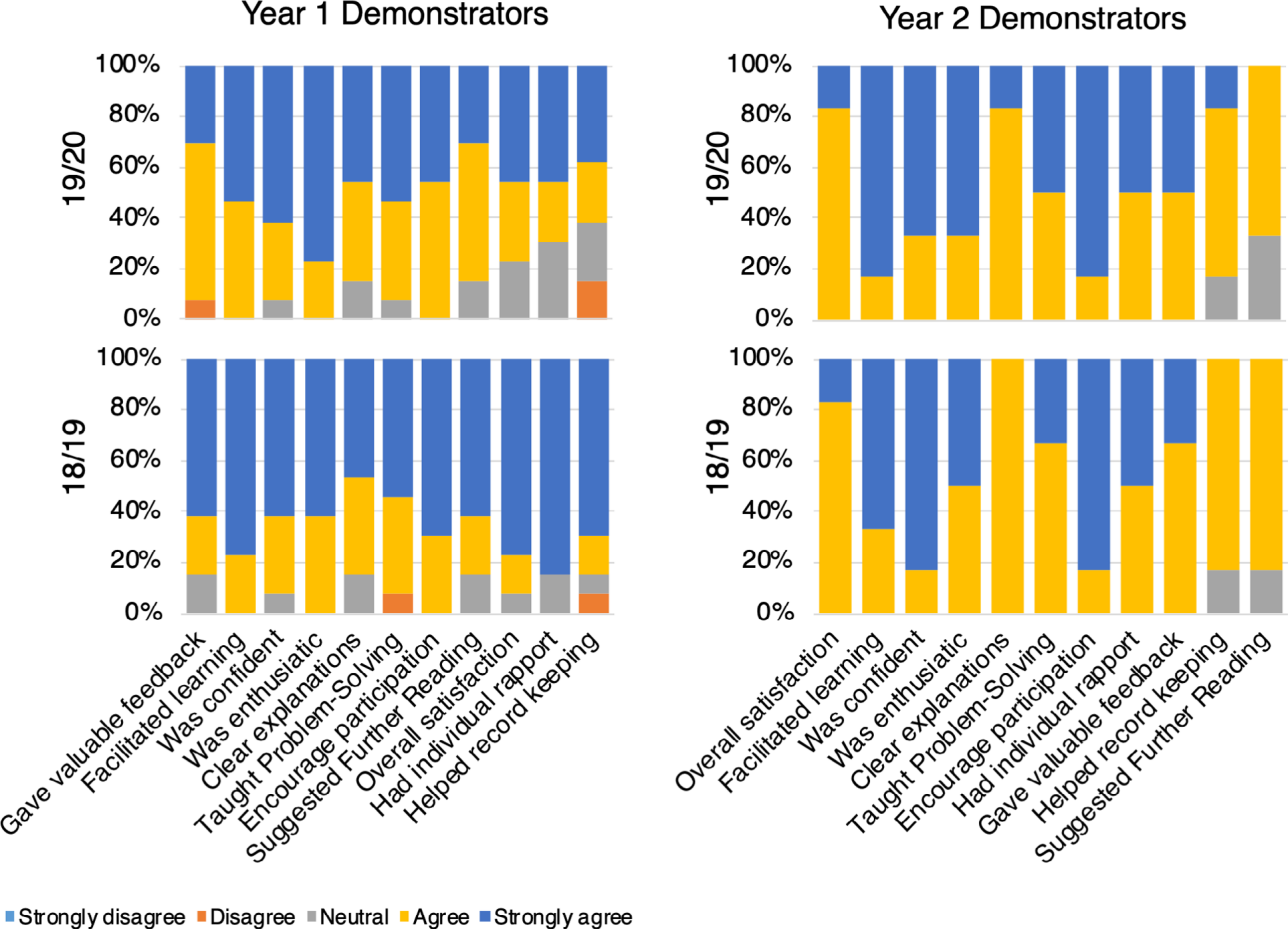
Self‐evaluation of demonstrators’ teaching effectiveness in 1^st^‐ and 2^nd^‐year practicals by 11 factor‐based SEEQ questionnaire following minimal training (201819, *n* = 76) and extensive training (201920, *n* = 283). Vertically paired columns for each factor are not significantly different (*P* > 0.05), and the % difference between 19/20 and 18/19 for strongly agree and agree categories combined is 0 for almost all factors.

In both first‐ and second‐year practicals, students scored demonstrators from 2019/2020 that had extensive training significantly higher in each of the 11 factors including ‘overall satisfaction’ (Mann–Whitney test, *P* ≤ 0.0001 (year 1), *P* ≤ 0.017 (year 2)) compared to demonstrators with minimal training from 2018/2019 (Figs. 1 and 2). Interestingly, one of the lowest rated areas in both years concerned the demonstrators’ ability to suggest further reading and suggests that they were too busy with other areas and this was still somewhat neglected. This was surprising as demonstrators were trained to point students to the reading lists in the laboratory manual and the online course site. This result indicates that there is a limit to what we can expect our demonstrators to do in the time they are trained and the time in the laboratory. As such, despite our increased two‐hour face‐to‐face weekly training sessions, some key areas would still need attention in the future.

It is clearly challenging to train demonstrators to improve all desired teaching skills within the dedicated face‐to‐face sessions. These sessions are best used for active training in helping students learn in the psychomotor and affective domains. As such, it is recommended to develop asynchronous training materials for cognitive domain training outside of these sessions. We also anticipate that providing instant feedback to demonstrators from academic staff within the laboratory will also help cover training that does not get covered in the dedicated sessions. This is consistent with other studies that find that in addition to separate training sessions, ‘on‐the‐job’ training is also crucial [15, 17].

### Demonstrators’ self‐evaluation of their teaching effectiveness

From the demonstrator self‐evaluation scores, we found that they rated themselves highly across all categories even when they received minimal training and no significant difference (*P* > 0.05) was observed after they received extensive training (Fig. 2). Interestingly however, in the comment section, 100% of demonstrators found the extensive training very useful compared with minimal training. The high scores with minimal training contradict the student opinion about their teaching ability. This shows that demonstrators’ perceptions about their teaching quality were different from students and were not able to fully anticipate student needs and adequately self‐reflect. If demonstrators are not aware of students’ expectations, then they may not be able to judge their own teaching effectiveness [8]. This ties in with Santhanam and Codner who said ‘an important concern about untrained teachers is that they do not concentrate on student learning, but instead concentrate on what they perceive they are expected to do’ [57]. These pre‐conceived and long held beliefs may determine *how* as well as *what* novice demonstrators learn about teaching [58]. Furthermore, lack of ability and time to reflect on their teaching effectiveness may also lead to these differing perceptions.

Based on these data, in the future, we will better integrate demonstrators’ self‐reflection into our training which has also been underscored in previous studies [17]. This could involve academic staff giving weekly feedback to demonstrators on their teaching. We would also expect demonstrators to keep a weekly reflective log as a record of their teaching progress which academic staff and demonstrators can discuss/review together.

### Decoding student satisfaction through thematic analysis

**Fig. 3:**
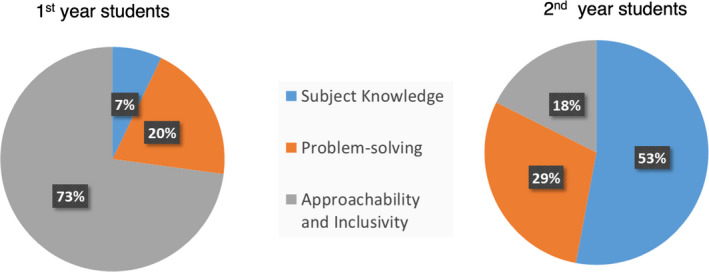
Pie charts based on the thematic analysis of the students’ responses in open comment section of questionnaire. The open text responses from 1^st^‐year (*n* = 70) and 2^nd^‐year (*n* = 102) students were coded to three main themes – subject knowledge, problem solving and approachability and inclusivity.

The open text students’ responses from the questionnaires indicated what mattered most to students about their demonstrators. These comments were associated with three main themes as shown in Table 4 and Fig. 3. 27% of first‐year students commented on demonstrators’ subject knowledge and problem‐solving skills, but the majority (73% students) commented on demonstrators’ approachability/inclusivity with over 94% of these comments being positive (Fig. S1). In these specific comments, students welcomed the ability of demonstrators to understand their individual needs, be receptive, patient and to provide them with an open and interactive learning environment. Interestingly, this was less important for 2nd‐year students where 82% students placed greater value on the provision of strong subject‐knowledge and problem‐solving skills (Fig. 3). Interestingly, over 86% of comments in these areas were positive (Fig. S1), which is also encouraging. Studies show that there is a change in students’ perspective as they transit from 1^st^ to 2^nd^ year of university education [27], which is consistent with the different challenges/goals each year group faces as discussed in the introduction. 1^st^‐year students are in process of exploring their identity; they are mainly focussed on academic and social integration and better appreciate pastoral support along the way. The 2^nd^‐year students, on the other hand, are further along this journey as they are predisposed to laboratory‐based learning and develop less dependency on others. They are also achievement driven and concentrate more on developing their academic identity [27]. Additionally, in 1^st^ year, bioscience students come from much more diverse academic backgrounds with different levels of preparation in maths, chemistry and biology. While in 2^nd^ year, students are from molecular bioscience programmes and many more will have taken chemistry and maths prior to starting university. These differences could also explain how students may have more independence in their learning in our 2^nd^‐year course.

### Research limitations

This study has a number of limitations. Based on demonstrator open comments, it is clear that the new training was useful and explains why the improvements were seen. However, since around 45% of our demonstrators were teaching for the second consecutive year, the improvements we have seen could be partly attributed to repeat teaching from this minority group, as this is known to also make a difference (evaluation scores improve on repeat) [15]. Also, the number of student respondents was lower in 2018 compared with 2019 as the surveys were distributed online in 2018 while they were distributed in person for 2019 which increased participation significantly. Finally, our improvements in student evaluations were significant; however, the study is based on only 2 years and it may have benefited from a longer time frame to ensure the trend is consistent, although this was not possible due to the large changes in teaching because of the COVID‐19 pandemic.

## Conclusions

Our studies have explored demonstrators’ training for bioscience students and revealed some key findings that further this field and are particularly applicable to laboratories with large cohort sizes.

### Demonstrators’ training in student feelings and their individual needs must not be neglected

Our training places an equal importance on developing demonstrators’ ability to understand students’ individual needs and to customize their teaching strategies to promote personalized student learning. This is particularly relevant during and after the COVID‐19 pandemic, which has taken its toll on school leaver’s mental health and confidence and who would find transitioning to university especially challenging and calls for training demonstrators to effectively deal with the academic unreadiness of 1st‐year students.

### Simultaneous student and demonstrator evaluation of teaching is a rich source of insights for demonstrator training

In our study, we have not only designed thorough strategies but also conducted student as well as demonstrators’ evaluation within a single study. This allowed quality assurance of our training programme and ensured productive learning on both sides as indicated by increase in teaching evaluation scores and/or comments. Furthermore, most SET‐based research studies are not multilevel as the comparisons are simply made between different disciplines. These uncontrollable variables due to different subjects pose limitations to these studies. Our study ran across two years and at two levels of bioscience in the same academic department. This direct and continuous comparison gave valuable insight about changing needs of students and how demonstrators’ training needs to be tailored. While both years require demonstrators to provide the essential psychomotor/cognitive instruction, 1^st^ years require more inclusive teaching approaches (affective domain) to give them confidence to become independent learners. Second‐year students, however, need more attention with their independent academic performance, such as problem‐solving skills (cognitive domain).

### The misalignment of demonstrators’ perceptions and students’ expectations suggests the need for demonstrator self‐reflection supported by academic staff

Simultaneous student and demonstrator student evaluations also allowed us to evidence the misalignment of demonstrators’ perceptions and students’ expectations. As such, ongoing reflection should be a critical component of training.

### Recommendations

**Fig. 4:**
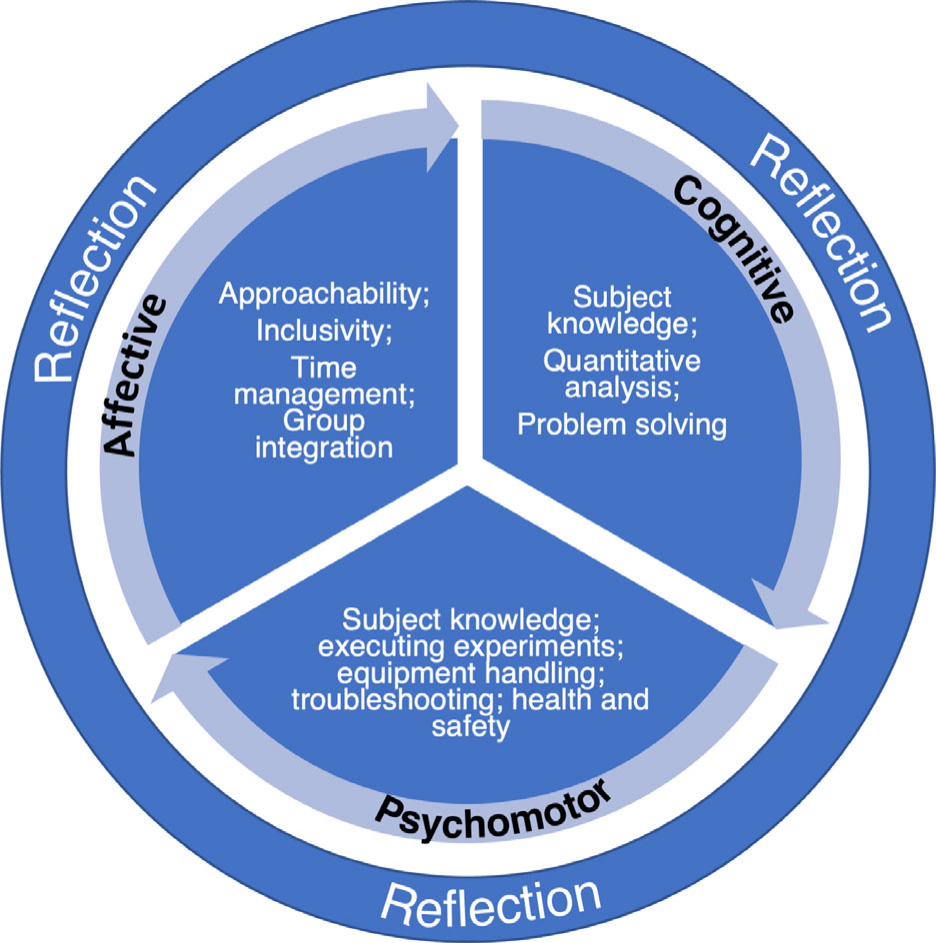
A multimodal demonstrator training framework that includes cognitive, psychomotor and affective domains as learning objectives of demonstrators’ training programme and places equal emphasis on each of the three domains. The training can be tailored as the students’ progress through their course of study, for example year 1 versus year 2. Demonstrators should be encouraged to engage in regular self‐reflection, and academic staff should facilitate this by giving regular feedback and discussion around their performance in these three domains.

Based on our findings, we present a multimodal training framework (Fig. 4) to guide course leaders’ demonstrator training strategy for their laboratories. The framework is a starting point for any practical module and includes the often‐overlooked student affective learning domain. The relative focus of each of the three Bloom’s learning domains (and activities within) does not necessarily need equal focus and can be adjusted according to the year level. To begin using this framework, we recommend considering these initial questions.

- Is there sufficient departmental budget available for demonstrators?
- Is there sufficient staff expertise to actively engage with demonstrators?
- Can face‐to‐face training be combined with asynchronous training resources to provide more flexibility?
- Can demonstrators be informed about the academic preparedness of their students (which will depend on year group) and is there a mechanism each week (such as a checklist/cheat sheet) to ensure they provide personalized student learning in their group?
- Can regular demonstrator self‐reflection be monitored, and staff feedback be provided?

This study and framework will allow us to better articulate to future demonstrators how they can positively impact laboratory‐based student learning and development over their first two years. With better understanding of active and student‐focussed training, we hope to better motivate demonstrators to create and sustain a learning environment that encourages and supports students’ efforts to learn.

## Conflict of interest

The authors declare no conflict of interest.

## Author contributions

RA conceived and designed the project, acquired 1^st^‐year data and wrote the paper. ES designed the project, acquired and analysed 2^nd^‐year data and wrote the paper.

## Supporting information


**Fig. S1**. In depth analysis of the students’ responses in open comments section of questionnaire. The open text responses from 1st year (*n* = 70) and 2nd year (*n* = 102) were coded to three main themes‐subject knowledge, problem solving and approachability & inclusivity (A&I). Within each category, the themes were split into positive or negative comments. The size of each bubble and the number within each bubble represent the percentage responses (calculated from total number of responses for years 1 and 2) in that category.

## Data Availability

All data not subject to limited distribution due to confidentiality agreements are available upon reasonable request to the first author, RA.
