# Supplementary figures and images for: Demonstrator training needs to be active and focused on personalized student learning in bioscience teaching laboratories
Source: FEBS Open Bio. 2021 Sep 27;11(11):2888–901. doi: 10.1002/2211-5463.13299 (PMC8564340; doi:10.1002/2211-5463.13299)

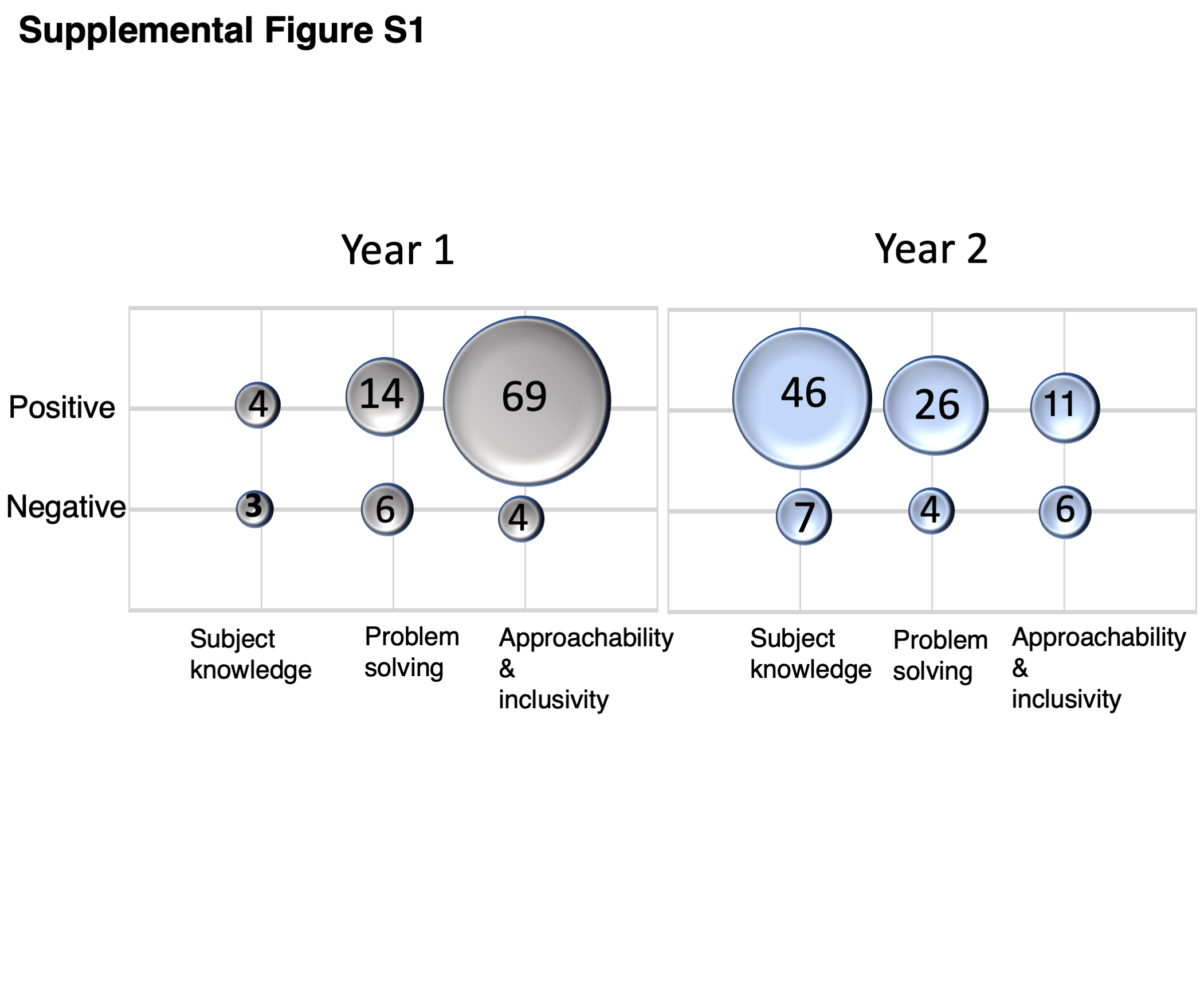

Supplement: Supplementary file 1 — Fig. S1. In depth analysis of the students’ responses in open comments section of questionnaire. The open text responses from 1st year (n = 70) and 2nd year (n = 102) were coded to three main themes‐subject knowledge, problem solving and approachability & inclusivity (A&I). Within each category, the themes were split into positive or negative comments. The size of each bubble and the number within each bubble represent the percentage responses (calculated from total number of responses for years 1 and 2) in that category. [file FEB4-11-2888-s001.tiff]
